# Pedestrian Positioning Using an Enhanced Ensemble Transform Kalman Filter

**DOI:** 10.3390/s23156870

**Published:** 2023-08-02

**Authors:** Kwangjae Sung

**Affiliations:** Department of Software, Sangmyung University, Cheonan-si 31066, Republic of Korea; kjsung@smu.ac.kr

**Keywords:** regional numerical weather prediction model, ensemble-based Kalman filter, state estimation, data assimilation

## Abstract

Due to the unavailability of GPS indoors, various indoor pedestrian positioning approaches have been designed to estimate the position of the user leveraging sensory data measured from inertial measurement units (IMUs) and wireless signal receivers, such as pedestrian dead reckoning (PDR) and received signal strength (RSS) fingerprinting. This study is similar to the previous study in that it estimates the user position by fusing noisy positional information obtained from the PDR and RSS fingerprinting using the Bayes filter in the indoor pedestrian positioning system. However, this study differs from the previous study in that it uses an enhanced state estimation approach based on the ensemble transform Kalman filter (ETKF), called QETKF, as the Bayes filer for the indoor pedestrian positioning instead of the SKPF proposed in the previous study. The QETKF estimates the updated user position by fusing the predicted position by the PDR and the positional measurement estimated by the RSS fingerprinting scheme using the ensemble transformation, whereas the SKPF calculates the updated user position by fusing them using both the unscented transformation (UT) of UKF and the weighting method of PF. In the field of Earth science, the ETKF has been widely used to estimate the state of the atmospheric and ocean models. However, the ETKF algorithm does not consider the model error in the state prediction model; that is, it assumes a perfect model without any model errors. Hence, the error covariance estimated by the ETKF can be systematically underestimated, thereby yielding inaccurate state estimation results due to underweighted observations. The QETKF proposed in this paper is an efficient approach to implementing the ETKF applied to the indoor pedestrian localization system that should consider the model error. Unlike the ETKF, the QETKF can avoid the systematic underestimation of the error covariance by considering the model error in the state prediction model. The main goal of this study is to investigate the feasibility of the pedestrian position estimation for the QETKF in the indoor localization system that uses the PDR and RSS fingerprinting. Pedestrian positioning experiments performed using the indoor localization system implemented on the smartphone in a campus building show that the QETKF can offer more accurate positioning results than the ETKF and other ensemble-based Kalman filters (EBKFs). This indicates that the QETKF has great potential in performing better position estimation with more accurately estimated error covariances for the indoor pedestrian localization system.

## 1. Introduction

The Global Navigation Satellite System (GNSS) has been widely used for the navigation of a person and a vehicle in outdoor environments, whereas its availability is highly limited due to the blocking of GNSS signals by walls and obstacles in indoor environments. To solve the unavailability issues of GNSS, various pedestrian positioning approaches that use deep learning techniques, inertial measurement units (IMUs), and wireless signals from radio beacons have been introduced.

To identify pedestrians in images, pedestrian detection algorithms based on deep learning techniques such as Convolutional Neural Network (CNN) [1] have been proposed in recent experiments and research [2,3,4,5]. The CNN is mainly classified into two categories: a two-stage framework and a one-stage framework. The two-stage framework that includes region-based CNN (R-CNN), fast R-CNN, and faster R-CNN [6] is executed with region proposal and object detection networks. In the two-stage framework, the input image is first processed to obtain region proposals. Then, CNN features are computed from interested regions, and the class of the object in the region proposals is determined using classifiers. The one-stage framework that includes You Only Look Once (YOLO) [7] and Single-Shot MultiBox Detector (SSD) [8] does not require a region proposal network by using a single feed-forward convolutional network. Compared to the two-stage framework, it consumes less computational cost by merging all computational steps into only one stage.

To track pedestrians in video streams, Simple Online and Real-Time Tracking (SORT) [9] for tracking multiple objects is widely used [10]. It estimates the position and velocity of objects using a Kalman filter and associates object detections across multiple frames of a video or image sequence using a Hungarian algorithm [11]. However, it has difficulty in tracking objects that are occluded. To solve this problem, Deep-SORT [12] associates object detections across multiple frames on the basis of appearance features of objects extracted from bounding box images through a separate CNN.

The pedestrian dead reckoning (PDR) as a localization method without additional infrastructures, such as GNSS and WiFi access points (APs), can locate the user by leveraging IMU sensors on the phone, including gyroscopes and accelerometers. The positioning procedure of the PDR can be divided into three parts: step detection, walking distance calculation, and heading angle estimation of the user (refer to Section 4.1 in [13]).

The acceleration readings from the accelerometer on the phone can be used to execute the step detection in the PDR system [14]. In the previous study [13], the user step was detected by analyzing peak acceleration values from the smartphone accelerometer sensor. The walking distance of the user can be calculated by empirical walking models obtained by analyzing the step frequency, acceleration values within a step, and angle between legs during a step [15]. In the previous study [13], the step length was estimated by analyzing the relationship between the step distance and acceleration value during a step. The walking direction angle of the user can be estimated by integrating the readings from the gyroscope and accelerometer [13,16]. The PDR can yield satisfactory positioning accuracy for short time intervals. However, since the PDR calculates the current location depending on the previous location, it can be prone to drift or cumulative error when the positioning is performed for a long time.

The received signal strength (RSS) fingerprinting is a positioning approach using RSS measurements from radio beacons, such as wireless fidelity (WiFi) access points [17], Bluetooth low energy (BLE) beacons [18], and cellular radio towers [19]. It first generates the fingerprint database (map) by collecting RSS values from radio beacons at positions where the localization is required and then estimates the current position of the user that best matches the measured RSS value from the fingerprint database.

Although the RSS fingerprinting method consumes a substantial amount of time to generate the RSS fingerprint map that represents the feature of the surrounding radio-frequency (RF) signals at a given position well, it generally does not require radio propagation models and positional information of radio beacons as in multilateration positioning techniques [20]. For this reason, the RSS fingerprinting method has been widely used for indoor positioning [21]. However, since the RF signal can be affected by multipath effect and obstacles as well as device deployment and heterogeneity, it can change over time or space, as shown in Figure 8 of Sung et al. [13]. Therefore, the RSS fingerprinting can result in inaccurate positioning results due to the characteristics of the RF signal.

To provide higher estimation accuracy in the positioning system, measurements from various sensors can be fused using Bayes filters, such as the particle filter (PF; Maohai et al. [22]), Kalman filter (KF; Chen et al. [23]), unscented Kalman filter (UKF; Zhan and Wan [24]), and ensemble-based Kalman filter (EBKF; Singh et al. [25]). For example, many indoor positioning systems based on the PF, KF, and UKF have been proposed to achieve better localization results for the user by integrating noisy positional information obtained from the PDR and RSS fingerprinting using the smartphone [23].

In the field of Earth science, the EBKFs, including the ensemble Kalman filter (EnKF; Evensen [26]), ensemble Kalman filter with perturbed observations (hereinafter EnKF-PO; Burgers et al. [27]), ensemble square root filter (EnSRF; Whitaker and Hamill [28]), and ensemble transform Kalman filter (ETKF; Bishop et al. [29]), have been widely used to estimate a large number of state variables of the atmospheric and ocean models.

The EBKF is similar to the PF in that it transforms a set of samples (also referred to as ensemble members) using the state transition model and then estimates the mean and error covariance of the state using transformed samples, thereby providing better estimation results than the KF and extended KF (EKF). However, while ensemble members in the EBKF are chosen using deterministic sampling based on the standard KF equations, those in the PF are determined by random sampling as in a Monte Carlo algorithm. For this reason, the EBKF generally requires a smaller number of samples than the PF for accurate and reliable state estimation.

Whereas the EBKF with the advantage of conducting efficient state estimation with a small and reasonable ensemble size has been widely used for atmospheric or ocean models, it is not a common method for position estimation in indoor environments. For example, Zhuang et al. [30] applied a smoother based on the EnKF to the visible light positioning (VLP) system using LED lights for mobile robot localization indoors.

Among the EBKF algorithms, the ETKF estimates the state of the model through the ensemble transformation using the posterior error covariance in ensemble space. Hence, it can perform more rapid state estimation than the EnKF, EnKF-PO, and EnSRF when a large number of observations are assimilated as in the atmospheric data assimilation [29].

However, the ETKF algorithm does not consider the model error in the state transition (or prediction) model. That is, it assumes a perfect model without any model errors [29]; generally, the model error is represented in the state transition model used for the indoor positioning system. Therefore, since the error covariance estimated by the ETKF does not include the model error covariance Q, it can be systematically underestimated. In addition, the state estimation performance of the ETKF can be degraded by underweighted observations due to the underestimated error covariance.

To solve this problem and to apply the ETKF to the indoor pedestrian localization system, this paper proposes an enhanced state estimation approach based on the ETKF (hereinafter QETKF), which estimates the error covariance where the model error covariance Q is included by considering the model error in the state prediction model.

**Figure 1:**
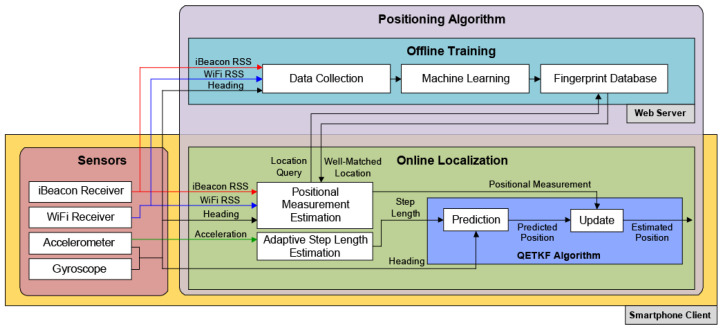
Overall architecture of the indoor positioning system that consists of the web server and smartphone. The indoor localization system in this study works using the sensors and the positioning algorithm in the same manner as that proposed by the previous study [13]; however, it differs from that of the previous study in that it uses the QETKF algorithm to estimate the user position in the online positioning phase instead of the SKPF algorithm proposed in the previous study.

This study is similar to the previous study [13] in that it estimates the user position by fusing noisy positional information obtained from the PDR and RSS fingerprinting using the Bayes filter in the indoor pedestrian positioning system. However, this study differs from the previous study [13] in that it uses the QETKF as the Bayes filer for the indoor pedestrian positioning instead of the SKPF proposed in the previous study. The contributions of this study are as follows:

- No indoor pedestrian positioning system based on the ETKF has been found to date, despite nearly a decade of searching. In this paper, an efficient approach for implementing the ETKF applied to the indoor pedestrian localization system that should consider the model error, which is called QETKF, is introduced. The QETKF is similar to the ETKF in that it is based on the ensemble transformation performed with the posterior error covariance in ensemble space, thereby providing more rapid positioning results than EnKF and its variants when many observations are assimilated as in the atmospheric data assimilation. However, the QETKF differs from the ETKF in that it can prevent the systematic underestimation of the error covariance that appears in the ETKF, since it can estimate the error covariances where the model error covariance Q is included by considering the model error in the prediction model, thereby producing more inflated ensemble spread than the ETKF. This enables the QETKF to yield higher estimation accuracy than the ETKF.
- The indoor localization system in this study is carried out using the sensors and the positioning algorithm as in the previous study [13]. In the localization system, the sensor part employs the IMU sensors (gyroscope and accelerometer) and wireless signal receivers (iBeacon and WiFi modules) on the smartphone in the same manner as the previous study [13]. The positioning algorithm part estimates the user position using the offline training and online positioning phases as in the previous study [13]. In the offline training phase, RSS data obtained from iBeacon and WiFi modules and user heading data obtained from the gyroscope and accelerometer on the smartphone are first collected at the positions predetermined for the localization indoors in the same manner as the previous study [13]. Then, the collected data (fingerprints) are transmitted to the web server and then transformed into the fingerprint database using the machine learning algorithm in the same manner as the previous study [13]. The difference between this study and the previous study is that this study utilizes the QETKF algorithm for the indoor positioning of users in the online positioning phase, whereas the previous study employs the SKPF, as shown in Figure 1. In the prediction step (i.e., PDR) of the online positioning phase, the QETKF predicts the user position with both the traveled distance of the user obtained from the adaptive step length estimation using the accelerometer and the heading information of the user obtained from the gyroscope and accelerometer on the smartphone as in the prediction step of the SKPF of the previous study [13]. In the update step of the online positioning phase, the QETKF first estimates the positional measurement using the RSS fingerprinting approach based on the machine learning algorithm without the help of the GNSS that provides considerably inaccurate localization information owing to the obstruction of the signals indoors, in the same manner as the update step of the SKPF of the previous study [13]. Then, the QETKF estimates the updated user location by fusing the predicted position by the prediction step (PDR) and the positional measurement estimated by the RSS fingerprinting scheme using the ensemble transformation, whereas the SKPF infers the updated user location by fusing them using both the unscented transformation (UT) of UKF and the weighting method of PF. The position estimation procedure of the QETKF using the ensemble transformation is addressed in more detail in Section 3.
- Similar to the previous study, pedestrian positioning experiments in this study were executed using the indoor localization system implemented on the smartphone in a campus building. However, unlike the previous study, the performance benefits of the QETKF applied to the indoor localization system were evaluated using the existing EBKFs as benchmarks, including the EnKF, EnKF-PO, EnSRF, and ETKF that have not been widely used for indoor pedestrian localization. Experimental results show that the QETKF can offer more accurate positioning results than the EnKF and ETKF and can accomplish positioning performance that is as accurate as the EnKF-PO and EnSRF. This indicates that the QETKF has great potential in carrying out better position estimation with more accurately estimated error covariances for the indoor pedestrian localization system. Furthermore, to examine the validity of the QETKF algorithm as an ensemble data assimilation method, the QEKTF was applied to the Lorenz-96 (L96) model [31], which is commonly used as a simple chaotic dynamical system in ensemble data assimilation. Data assimilation experimental results executed using the L96 model and many observation variables indicate that the QEKTF can provide higher state estimation accuracy than other EBKFs while consuming less computational time compared to the EnKF, EnKF-PO, and EnSRF.

The rest of this paper is organized as follows: Section 2 presents a summary of related work; Section 3 introduces the QETKF and the indoor localization system used in this study; Section 4 shows experiments and discussion to verify the estimation performance of the QETKF applied to the indoor localization system and the L96 model; and finally, Section 5 summarizes experimental results and discusses the potential of the QETKF for indoor localization.

## 2. Related Work

### 2.1. EBKF

In the field of Earth science, the EBKFs, including the EnKF, EnKF-PO, EnSRF, and ETKF, have been widely used to estimate the state of the atmospheric and ocean models capturing observation information in an effective manner.

The EnKF [26] propagates a set of ensemble members using the state transition (or prediction) model and then estimates flow-dependent prior error covariances of the model state using propagated ensemble members, thereby providing better estimation results than the KF and EKF. However, the posterior error covariance in the EnKF is systematically underestimated, owing to the inaccurate calculation for the posterior error covariance [27]. Accordingly, the estimation performance of the EnKF can be degraded, since observations are underweighted due to the systematic underestimation of the posterior error covariance.

The EnKF-PO [27] deals with perturbed observations as random variables to solve the problem of the systematic underestimation of the error covariance appearing in the EnKF. However, the additional sampling error relevant to perturbed observations can lead to inaccurate estimation results due to the systematic underestimation (biased estimation) of the error covariance calculated by the EnKF-PO when a small number of ensemble members are used [28].

Unlike the EnKF-PO, the EnSRF [28] utilizes the reduced Kalman gain to deal with the issue of the systematic underestimation of the posterior error covariance appearing in the EnKF. Therefore, it does not lead to an extra source of sampling error associated with perturbed observations as in the EnKF-PO, thus providing lower estimation error than the EnKF-PO for a small ensemble size.

The ETKF [29] uses the ensemble transformation to resolve the issue of the systematic underestimation of the error covariances appearing in the EnKF and EnKF-PO. However, the ETKF does not take into consideration the model error $w_{k}$ in the state transition (or prediction) model as in Equation (1). Therefore, since the error covariance estimated by the ETKF does not include the model error covariance $Q_{k}$, it can be systematically underestimated. Moreover, the state estimation performance of the ETKF can be degraded by underweighted observations due to the underestimated error covariance.

### 2.2. ETKF

Among the EBKF algorithms, the ETKF algorithm [29] and its variants, such as local ensemble transform Kalman filter (LETKF; Hunt et al. [32]), that are most widely used in the field of Earth science estimate the state $x_{k}$ of the nonlinear system that consists of both the prediction model f(·) without additive model error and the observation operator h(·) with additive observation error $v_{k}$ with mean zero and covariance matrix $R_{k}$ at time *k*, as follows:

(1) $$x_{k}=f\left(x_{k-1}\right)$$

(2) $$z_{k}=h\left(x_{k}\right)+v_{k}.$$

The nonlinear model f(·) is used to propagate the system state $x_{k-1}$ at time k−1 to the next estimation time *k*, and the observation operator h(·) determines the observation $z_{k}$ in observation space for the system state $x_{k}$ in model space by mapping from model space to observation space.

The state estimation procedure of the ETKF can be classified into two phases: the prediction phase that performs ensemble forecasts using the prediction model f(·) and the update phase that assimilates observations using the observation operator h(·).

#### 2.2.1. Prediction (Ensemble Forecast)

During this phase, a prior or predicted ensemble ${\left\{x_{k|k-1}^{i}\right\}}_{i=1}^{n}$ of *m*-dimensional model state vectors at time *k* can be obtained by transforming a posterior ensemble ${\left\{x_{k-1|k-1}^{i}\right\}}_{i=1}^{n}$ at time k−1 to the next estimation time *k* through the prediction model f(·) (i.e., ensemble forecast):

(3) $$x_{k|k-1}^{i}=f\left(x_{k-1|k-1}^{i}\right)\;\;\;\mathrm{for}\;\;\;i=1,\ldots ,n.$$

Using the prior ensemble ${\left\{x_{k|k-1}^{i}\right\}}_{i=1}^{n}$, the prior ensemble mean ${\hat{x}}_{k|k-1}$ and its error covariance $P_{k|k-1}$ can be calculated by

(4) $${\hat{x}}_{k|k-1}=n^{-1}\sum_{i=1}^{n}x_{k|k-1}^{i}$$

(5) $$P_{k|k-1}={\left(n-1\right)}^{-1}X^{b}{\left(X^{b}\right)}^{T}$$

where $X^{b}$ is the m×n prior ensemble perturbation matrix whose *i*th column vector is $x_{k|k-1}^{i}-{\hat{x}}_{k|k-1}$.

#### 2.2.2. Update (Observation Assimilation)

The observation operator h(·) that executes the mapping from model space to observation space is used to obtain a prior observation ensemble ${\left\{z_{k}^{i}\right\}}_{i=1}^{n}$ of *l*-dimensional observation vectors for the prior ensemble ${\left\{x_{k|k-1}^{i}\right\}}_{i=1}^{n}$ in model space at time *k* as follows:

(6) $$z_{k}^{i}=h\left(x_{k|k-1}^{i}\right)\;\;\;\mathrm{for}\;\;\;i=1,\ldots ,n.$$

Using the prior ensemble ${\left\{z_{k}^{i}\right\}}_{i=1}^{n}$ in observation space, the prior observation ensemble mean ${\hat{z}}_{k}$ and its corresponding error covariance $S_{k}$ can be calculated as

(7) $${\hat{z}}_{k}=n^{-1}\sum_{i=1}^{n}z_{k}^{i}$$

(8) $$S_{k}={\left(n-1\right)}^{-1}Y^{b}{\left(Y^{b}\right)}^{T}+R_{k}$$

where $Y^{b}$ is the l×n prior observation ensemble perturbation matrix whose *i*th column vector is $z_{k}^{i}-{\hat{z}}_{k}$.

In the ETKF, the optimal Kalman gain $K_{k}$, posterior or updated ensemble mean ${\hat{x}}_{k|k}$, and its error covariance $P_{k|k}$ can be calculated using ensemble perturbation matrices $X^{b}$ and $Y^{b}$, as in [29,32]:

(9) $$K_{k}=X^{b}{\tilde{P}}^{a}{\left(Y^{b}\right)}^{T}R_{k}^{-1}$$

(10) $${\hat{x}}_{k|k}={\hat{x}}_{k|k-1}+K_{k}\left(z_{k}-{\hat{z}}_{k}\right)$$

(11) $$P_{k|k}=X^{b}{\tilde{P}}^{a}{\left(X^{b}\right)}^{T}$$

where ${\tilde{P}}^{a}$ denotes the n×n posterior error covariance in ensemble space and is given by

(12) $${\tilde{P}}^{a}={\left[\left(n-1\right)I+{\left(Y^{b}\right)}^{T}R_{k}^{-1}Y^{b}\right]}^{-1}.$$

Similar to the prior error covariance $P_{k|k-1}$ in Equation (5), the posterior error covariance $P_{k|k}$ given by Equation (11) can be rewritten using the posterior ensemble perturbation matrix $X^{a}$ whose *i*th column vector is $x_{k|k}^{i}-{\hat{x}}_{k|k}$ as follows:

(13) $$P_{k|k}={\left(n-1\right)}^{-1}X^{a}{\left(X^{a}\right)}^{T}.$$

From Equations (11) and (13), the posterior ensemble perturbation matrix $X^{a}$ can be written as

(14) $$X^{a}=X^{b}\sqrt{\left(n-1\right){\tilde{P}}^{a}}.$$

That is, the prior ensemble perturbation matrix $X^{b}$ is transformed into the posterior ensemble perturbation matrix $X^{a}$ using Equation (14) (hence the “ensemble transform” in the name ETKF).

From Equations (10) and (14), the posterior ensemble members ${\left\{x_{k|k}^{i}\right\}}_{i=1}^{n}$ at time *k* in model space can be determined by

(15) $$x_{k|k}^{i}={\hat{x}}_{k|k}+X^{a(i)}\;\;\;\mathrm{for}\;\;\;i=1,\ldots ,n$$

where $X^{a(i)}$ denotes the *i*th column vector of $X^{a}$.

### 2.3. Model Error Issues with ETKF

The ETKF algorithm does not take into consideration the model error $w_{k}$ in the state transition (or prediction) model, as in Equation (1); that is, it assumes a perfect model without any model errors. Hence, the model error covariance $Q_{k}$ is not represented in the prior error covariance $P_{k|k-1}$ estimated by the ensemble members in the ETKF, as in Equation (5). This is because the basic idea behind the ETKF assumes that if the ensemble forecasts are made with differing but similarly skillful prediction models, the prior error covariance $P_{k|k-1}$ will implicitly include flow-dependent estimates for the model error covariance $Q_{k}$ [29]. Excluding the model error covariance $Q_{k}$ from Equation (5) enables the ETKF to more rapidly execute the state estimation through the ensemble transformation given by Equation (14) that uses the posterior error covariance ${\tilde{P}}^{a}$ in ensemble space, compared to the EnKF and its variants. However, it can result in the systematic underestimation (i.e., biased estimation) of the covariance $P_{k|k-1}$, especially when a small ensemble size is used due to the sampling error [33]. Consequently, the state estimation performance of the ETKF can be degraded by underweighted observations [34].

For the EBKFs, the most common approach to addressing model errors is to increase the size of the ensemble distribution around the ensemble mean (i.e., ensemble spread) using covariance inflation methods, including additive inflation, multiplicative inflation, and relaxation algorithms, which provide a possible solution to issues related to the underestimation of ensemble error covariances $P_{k|k-1}$ and $P_{k|k}$.

Assuming that model error $w_{k}$ is white noise with the zero-mean and covariance matrix $Q_{k}$ as in the standard KF equations, the additive inflation method [33] increases the ensemble spread in the EBKFs by adding random model error generated from the prescribed model error covariance $Q_{k}$ to ensemble members. However, the random model error used in the additive inflation method needs to be flow-dependent additive noise with rapid growth rates for subsequent use in the EBKF-based estimation system, especially for atmospheric and ocean applications [33].

For the multiplicative covariance inflation method, the inflated background error covariance can be obtained by multiplying the background error covariance $P_{k|k-1}$ by an inflation parameter. Generally, a large constant value of the inflation parameter can lead to an excessive covariance growth during recursive estimation procedures. To resolve the issue, adaptive multiplication inflation methods [35] have been designed for EBKF-based state estimation systems.

In the ETKF algorithm, since the posterior ensemble perturbations $X^{a}$ are influenced by model errors and are also calculated using Equation (14), they can be underdispersive, especially when a large number of observations are assimilated in the state estimation [34,35]. To deal with this problem, ad hoc methods that relax the reduction of the posterior ensemble perturbations $X^{a}$ have been introduced, such as the relaxation to prior perturbation (RTPP; Zhang et al. [36]) and the relaxation to prior spread (RTPS; Whitaker and Hamill [34]).

After the state estimation is executed using observations, the RTPP method can inflate the underestimated posterior perturbations $X^{a}$ back to the prior ensemble perturbations $X^{b}$:

(16) $$X_{\mathrm{RTPP}}^{a}=\alpha_{\mathrm{RTPP}}X^{b}+\left(1-\alpha_{\mathrm{RTPP}}\right)X^{a}$$

where $X_{\mathrm{RTPP}}^{a}$ are the posterior ensemble perturbations inflated by the RTPP and $\alpha_{\mathrm{RTPP}}$ is the inflation parameter of the RTPP. The parameter $\alpha_{\mathrm{RTPP}}$ should be set to a small positive value ($0\leq \alpha_{\mathrm{RTPP}}\leq 1$).

In a manner analogous to the RTPP given by Equation (16), the RTPS algorithm relaxes the posterior ensemble spread $\sigma^{a}$ back to the prior ensemble spread $\sigma^{b}$ as follows:

(17) $$\sigma_{\mathrm{RTPS}}^{a}=\alpha_{\mathrm{RTPS}}\sigma^{b}+\left(1-\alpha_{\mathrm{RTPS}}\right)\sigma^{a}$$

where $\sigma_{\mathrm{RTPS}}^{a}$ is the posterior ensemble spread inflated by the RTPS and $\alpha_{\mathrm{RTPS}}$ is the inflation parameter of the RTPS ($0\leq \alpha_{\mathrm{RTPS}}\leq 1$). The ensemble spreads $\sigma^{a}$ and $\sigma^{b}$ can be calculated by

(18) $$\sigma^{a}=\sqrt{{\left(n-1\right)}^{-1}X^{a}{\left(X^{a}\right)}^{T}}$$

(19) $$\sigma^{b}=\sqrt{{\left(n-1\right)}^{-1}X^{b}{\left(X^{b}\right)}^{T}}.$$

To inflate the underdispersive posterior perturbations $X^{a}$ using the inflated posterior spread $\sigma_{\mathrm{RTPS}}^{a}$, another inflation parameter $\beta_{\mathrm{RTPS}}$ for the RTPS can be calculated as

(20) $$\begin{array}{cc} \beta_{\mathrm{RTPS}} & =\frac{\sigma_{\mathrm{RTPS}}^{a}}{\sigma^{a}}\\ & =\alpha_{\mathrm{RTPS}}\frac{\sigma^{b}-\sigma^{a}}{\sigma^{a}}+1. \end{array}$$

Multiplying the inflation parameter $\beta_{\mathrm{RTPS}}$ by the posterior perturbations $X^{a}$, the RTPS can determine the inflated posterior perturbations $X_{\mathrm{RTPS}}^{a}$ as follows:

(21) $$X_{\mathrm{RTPS}}^{a}=\beta_{\mathrm{RTPS}}X^{a}.$$

The RTPP can inflate the posterior ensemble perturbations while preserving the growing characteristics of the prior ensemble perturbations $X^{b}$ during the prediction phase, as in Equation (16). For this reason, the RTPP with a large value of $\alpha_{\mathrm{RTPP}}$ can induce the overdispersion of the posterior ensemble perturbations [37]. On the contrary, the relaxed posterior perturbations $X_{\mathrm{RTPS}}^{a}$ obtained by the RTPS are less sensitive to the prior ensemble perturbations $X^{b}$, as in Equation (21).

## 3. Methodology

### 3.1. QETKF

The prediction and update phases of the QETKF are carried out in the same manner as those of the ETKF. However, unlike the ETKF, the QETKF takes into consideration the model error $w_{k}$ in the state transition model; therefore, the prediction model f(·) in the QETKF can be expressed as

(22) $$x_{k}=f\left(x_{k-1}\right)+w_{k}.$$

Using Equation (22), the prior and posterior ensemble error covariances $P_{k|k-1}^{Q}$ and $P_{k|k}^{Q}$ used in the QETKF can be written as (more details for the derivation of $P_{k|k}^{Q}$ can be found in Appendix A):

(23) $$P_{k|k-1}^{Q}={\left(n-1\right)}^{-1}X^{b}{\left(X^{b}\right)}^{T}+Q_{k}$$

(24) $$P_{k|k}^{Q}=X^{b}{\tilde{P}}^{a}{\left(X^{b}\right)}^{T}+Q_{k}.$$

Unlike Equations (5) and (11) used in the ETKF, the model error covariance $Q_{k}$ is included in the error covariances $P_{k|k-1}^{Q}$ and $P_{k|k}^{Q}$ produced by the QETKF, as in Equations (23) and (24). This allows the QETKF to avoid the systematic underestimation of the error covariances calculated by the ETKF, as in Equations (5) and (11). Consequently, the QETKF can provide better estimation accuracy than the ETKF by using the inflated error covariances, as if the covariance inflation method is applied.

The posterior ensemble members ${\left\{x_{k|k}^{i}\right\}}_{i=1}^{n}$ can be determined using Equations (15), (18), and (19) as follows:

(25) $$x_{k|k}^{i}={\hat{x}}_{k|k}+\left(\alpha +\beta \frac{\sigma^{b}-\sigma^{a}}{\sigma^{a}}\right)X^{a(i)}.$$

The parameter α in Equation (25) is given by

(26) $$\alpha =\sqrt{\frac{\mathrm{trace}\left(P_{k|k}^{Q}\right)}{\mathrm{trace}\left(P_{k|k}\right)}}.$$

This parameter ensures that the posterior error variance estimated by the ETKF approximates that estimated by the QETKF.

As mentioned above, the posterior ensemble perturbations $X^{a}$ can be underdispersive by Equation (14) when the observation is assimilated [34,35]; that is, the error covariances $P_{k|k-1}$ and $P_{k|k}$ estimated by the QETKF can be systematically underestimated. To resolve this problem, the tunable parameter β (0≤β≤1) in Equation (25) is used to inflate the ensemble perturbations $X^{a}$ in a manner similar to the RTPS given by Equation (21).

### 3.2. Indoor Positioning System

The development and testing of the indoor positioning system used in this study are based on the localization system that consists of the web server and smartphone, which was originally developed by the previous study [13], as shown in Figure 1.

In this study, the web server used for the indoor positioning system is equipped with two 2.0 GHz Intel Xeon Gold 6338 processors, each of which is equipped with 32 CPU cores and uses 64 GB of random access memory, running on CentOS Linux. In addition, the web server in this study works in the same way as that in the previous study [13]; that is, it receives location queries related to the current location estimation from the user phone and carries out the machine learning approach for the indoor localization. The phone client (iPhone 11) collects sensory readings from built-in sensors and then uses them to localize the user, as in the previous study [13].

The indoor localization system in this study works using the sensors and the positioning algorithm as in the previous study [13]. In the localization system, the sensor part leverages the IMU sensors (gyroscope and accelerometer) and wireless signal receivers (iBeacon and WiFi modules) on the phone in the same manner as the previous study [13].

For the positioning algorithm part, the user position is estimated by the offline training and online positioning phases, as in the previous study [13]. In the offline training phase for the site survey, the RSS data obtained from iBeacon and WiFi modules as well as user heading values obtained from the gyroscope and accelerometer on the phone are first corrected at the positions predetermined for the localization indoors in the same manner as the previous study [13]. Then, the collected data (fingerprints) are sent to the web server and then converted into the fingerprint database using the machine learning algorithm based on the naive Bayes classifier (NBC) in the same manner as the previous study [13].

The difference between this study and the previous study is that this study uses the QETKF algorithm for the indoor localization of users in the online positioning phase, while the previous study uses the SKPF algorithm, as shown in Figure 1. In the online localization phase, the QETKF estimates the user location using the prediction and update steps that are based on the pedestrian model introduced in previous study [13], which describes the motion of the user with the smartphone. More details on the pedestrian model can be found in Section 4.3 of Sung et al. [13].

For the prediction step (i.e., PDR) of the QETKF, the predicted user location ${\hat{x}}_{k|k-1}$ is computed with the traveled distance of the user obtained by the adaptive step length estimation using the accelerometer as well as the heading information of the user obtained by the gyroscope and accelerometer on the smartphone as in the prediction step of the SKPF of the previous study [13]. A more detailed description for the adaptive traveled distance estimation and the heading determination of the user can be found in Section 4.1 of Sung et al. [13].

During the update step, the QETKF first infers the positional measurement $z_{k}$ using the fingerprinting approach based on the machine learning algorithm (i.e., NBC) instead of the GNSS that provides considerably inaccurate localization information owing to the obstruction of the signals indoors, in the same manner as the update step of the SKPF of the previous study [13]. More details on the positional measurement estimation by the fingerprinting scheme based on the machine learning can be found in Section 4.2 of Sung et al. [13]. Then, the QETKF estimates the updated user position ${\hat{x}}_{k|k}$ by fusing the predicted position ${\hat{x}}_{k|k-1}$ by the prediction step (PDR) and the positional measurement $z_{k}$ estimated by the NBC-based fingerprinting scheme using the ensemble transformation, while the SKPF computes the corrected user position ${\hat{x}}_{k|k}$ by fusing them using both the unscented transformation (UT) of UKF and the weighting method of PF. The position estimation procedure of the QETKF using the ensemble transformation is addressed in more detail in Section 3.3.

### 3.3. QETKF-Based Localization Algorithm

Among the EBKF algorithms, the ETKF with the advantage of conducting efficient state estimation with a small and reasonable ensemble size through the ensemble transformation has been widely used for atmospheric or ocean models. However, since the ETKF does not consider the model error in the state transition (or prediction) model, the error covariance estimated by it does not include the model error covariance Q and thus can be systematically underestimated. In addition, the ETKF is not a common method for position estimation in indoor environments. In this paper, the QETKF, which is an efficient approach for implementing the ETKF applied to the indoor pedestrian localization system that should consider the model error, is proposed.

Similar to the ETKF, the QETKF estimates the state of the model through the ensemble transformation using the posterior error covariance in ensemble space. Therefore, it can provide more rapid positioning results than the EnKF, EnKF-PO, and EnSRF when many observations are assimilated as in the atmospheric data assimilation.

Unlike the ETKF, the QETKF can prevent the systematic underestimation of the error covariance appearing in the ETKF since it can estimate the error covariances where the model error covariance Q is included by considering the model error in the prediction model. Thus, the QETKF can apply the ensemble transformation of the ETKF to the state estimation of the indoor pedestrian model with the model error. Moreover, the QETKF can produce more inflated ensemble spread than the ETKF by solving the problem with the systematic underestimation of the error covariance appearing in the ETKF. This enables the QETKF to yield higher estimation accuracy than the ETKF.

In this study, the movement of the pedestrian for indoor environments can be described by the pedestrian model in Section 4.3 of Sung et al. [13]. In the pedestrian model, the state (i.e., position information) of the pedestrian can be expressed by the vector $x_{k}={\left[\begin{array}{cc} x_{k} & y_{k} \end{array}\right]}^{T}$, where $x_{k}$ and $y_{k}$ denote *x*-axis and *y*-axis coordinates in the navigation frame, respectively.

Algorithm 1 represents the indoor localization approach based on the QETKF using the pedestrian model that consists of the matrices $F_{k-1}$ and $H_{k}$. In the prediction (ensemble forecast) phase, the prior or predicted position ${\hat{x}}_{k|k-1}$ of the user and its corresponding error covariance $P_{k|k-1}^{Q}$ at time *k* are calculated by propagating the posterior ensemble ${\left\{x_{k-1|k-1}^{i}\right\}}_{i=1}^{n}$ at time k−1 using 2×2 identity matrix $F_{k-1}$, vector $G_{k-1}={\left[\begin{array}{cc} \sin(\gamma ) & \cos(\gamma ) \end{array}\right]}^{T}$, user’s traveled distance *d* between times k−1 and *k* (refer to Section 4.1.1 in Sung et al. [13]), and user’s heading angle γ at time *k* (refer to Section 4.1.2 in Sung et al. [13]).
**Algorithm 1** QETKF-based indoor positioning approach$\left[{\left\{x_{k|k}^{i}\right\}}_{i=1}^{n}\right]=\mathrm{QETKF}\left[{\left\{x_{k-1|k-1}^{i}\right\}}_{i=1}^{n},z_{k}\right]$Calculate ensemble members, ensemble mean, and its error covariance• Prediction (ensemble forecast)  · Determine the prior ensemble ${\left\{x_{k|k-1}^{i}\right\}}_{i=1}^{n}$ by transforming the posterior ensemble     ${\left\{x_{k-1|k-1}^{i}\right\}}_{i=1}^{n}$ at time k−1 to the next time *k* using (6) in [13]     **for** i=1:n **do**      $x_{k|k-1}^{i}=F_{k-1}x_{k-1|k-1}^{i}+G_{k-1}d$     **end for**  · Compute the prior ensemble mean ${\hat{x}}_{k|k-1}$ and its error covariance $P_{k|k-1}^{Q}$ using (4)   and (23)• Update (observation assimilation)  · Determine the prior ensemble ${\left\{z_{k}^{i}\right\}}_{i=1}^{n}$ in observation space using (7) in [13]      **for** i=1:n **do**        $z_{k}^{i}=H_{k}x_{k|k-1}^{i}$      **end for**  · Compute the ensemble perturbations $X^{b}$ in (5) and $Y^{b}$ in (8)  · Compute the posterior error covariance ${\tilde{P}}^{a}$ in ensemble space using (12)  · Determine the Kalman gain $K_{k}$, posterior ensemble mean ${\hat{x}}_{k|k}$, error covariance $P_{k|k}$,    ensemble perturbation $X^{a}$, and inflated error covariance $P_{k|k}^{Q}$ using (9)–(11), (14),    and (24), respectively  · Determine the posterior ensemble ${\left\{x_{k|k}^{i}\right\}}_{i=1}^{n}$ by the ensemble sampling using (25)

During the update (observation assimilation) phase, the posterior or updated position ${\hat{x}}_{k|k}$ of the user, error covariances $P_{k|k}$ and $P_{k|k}^{Q}$, and ensemble perturbation $X^{a}$ are calculated by the predicted position ${\hat{x}}_{k|k-1}$ of the user, 2×2 identity matrix $H_{k}$, positional observation $z_{k}$ estimated by the NBC-based fingerprinting scheme (refer to Section 4.2 in Sung et al. [13]), and the posterior error covariance ${\tilde{P}}^{a}$ in ensemble space.

The QETKF that uses the posterior ensemble ${\left\{x_{k|k}^{i}\right\}}_{i=1}^{n}$ obtained by Equation (25) can produce more inflated posterior ensemble spread than the ETKF; that is, it can avoid the systematic underestimation of the posterior error covariance appearing in the ETKF, thus providing better positioning results. The positioning performance of the QETKF is discussed in more detail in Section 4.

## 4. Results and Discussion

### 4.1. Indoor Pedestrian Positioning Experiments

#### 4.1.1. Experimental Settings

To examine the performance of the indoor positioning scheme based on the KF and EBKFs, empirical experiments are carried out for two testbeds as in Sung et al. [13]: Scenarios S1 and S2. Scenario S1 represents the testbed with good radio conditions in the lecture room where RF signals are not frequently blocked by walls and obstacles. On the contrary, Scenario S2 represents the testbed with bad radio conditions in the hallways where RF signals are frequently blocked by walls and obstacles. More details on the testbeds can be founded in Section 5 of Sung et al. [13].

For analyzing the localization performance, fifty users with smartphones where the location estimation scheme is implemented walked along positions represented by a sequence number in Scenarios S1 and S2 clockwise (i.e., orange circle symbols (Scenario S1) and green square symbols (Scenario S2) shown in Figure 7 of Sung et al. [13]). Then, their locations were inferred by the KF or EBKFs in the positioning scheme.

Table 1 represents notations and features of localization approaches used for experiments. More details of localization approaches can be found in Section 6.2 of Sung et al. [13]. For the positioning approach P (i.e., PDR), the location of the pedestrian is predicted by sensory information (direction and acceleration) measured by IMU devices (accelerometer and gyroscope) in mobile phones (refer to Section 4.1 in [13]). Without the help of the GNSS, observations for user positions can be obtained from the NBC-based fingerprinting scheme in localization approaches (refer to Section 4.2 in [13]). However, the user location information and observation determined by the position prediction approach P and fingerprinting scheme may have drift error and large bias.

When the KF and EBKFs are applied to localization methods, the pedestrian location can be corrected by fusing positional data and observation with uncertainty that are gained by P and the fingerprinting scheme. The localization methods where the KF and EBKFs are applied can be categorized into three operational modes according to the type of training information (user heading, WiFi RSS, and iBeacon RSS) used in the fingerprinting approach: PU1, PU2, and PU3. The positioning approaches PU1, PU2, and PU3 predict the user position using the localization approach P. Then, the predicted position of the user is updated by the positional observation $z_{k}$ obtained from the NBC-based fingerprinting approach using user direction and WiFi RSS values for PU1, using user direction and iBeacon RSS values for PU2, and using user heading, WiFi RSS, and iBeacon RSS values for PU3.

For the QETKF algorithm, the choice of the parameter β addressed in Section 3.1 has an impact on the positioning performance. For all the experiments performed in this study, the tunable parameter β for the QETKF was set to 0.4. Positioning experiments (not shown here) showed that the QETKF with β=0.4 can offer accurate and reliable localization results in this study. As the value of β is increased above the optimal value, the QETKF cannot offer better positioning performance because the error covariances $P_{k|k-1}$ and $P_{k|k}$ estimated by the QETKF are increasingly overestimated. As the value of β is decreased to 0, the positioning performance of the QETKF is degraded by underweighted observations. This is because the posterior ensemble perturbations $X^{a}$ can be underdispersive by Equation (14) when the observation is assimilated, as mentioned in Section 3.1. That is, when the value of β is closer to 0, the error covariances $P_{k|k-1}$ and $P_{k|k}$ estimated by the QETKF can be systematically underestimated. A more detailed description of sensitivities to the parameter is beyond the scope of this study.

#### 4.1.2. Experiment with Ensemble Size and Positioning Scheme

**Figure 2:**
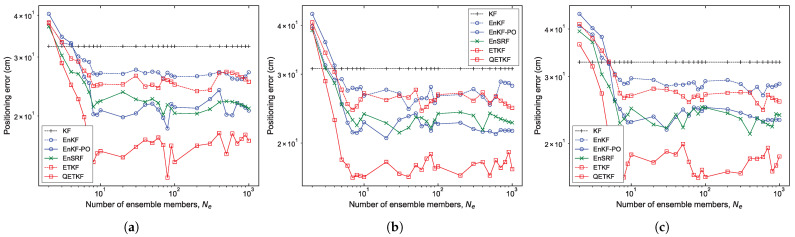
Positioning error of the KF, EnKF, EnKF-PO, EnSRF, ETKF, and QETKF as a function of ensemble size $N_{e}$ with respect to positioning schemes (**a**) PU1, (**b**) PU2, and (**c**) PU3 in Scenario S1.

**Figure 3:**
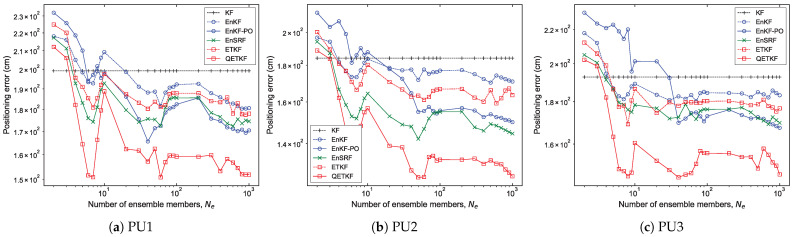
As in Figure 2, but for Scenario S2.

Figure 2 and Figure 3 show the average values of the positioning error of the KF, EnKF, EnKF-PO, EnSRF, ETKF, and QETKF according to ensemble size $N_{e}$ and positioning schemes PU1, PU2, and PU3 in Scenarios S1 (Figure 2) and S2 (Figure 3) during experiments executed by fifty pedestrians. On average, the ensemble-based Kalman filters (EBKFs), including the EnKF, EnKF-PO, EnSRF, ETKF, and QETKF, offered stable filtering solutions when $N_{e}\geq 8$ for Scenario S1 (Figure 2) and $N_{e}\geq 40$ for Scenario S2 (Figure 3); that is, their positioning error values decreased abruptly until the ensemble size $N_{e}$ reached 8 for Scenario S1 and 40 for Scenario S2 (i.e., the optimal ensemble size) and then converged to any value. The use of excessive ensemble members makes the EBKFs computationally unfeasible while providing more accurate estimation results.

It is shown in the figures that the KF that is not affected by sampling error surpasses the positioning accuracy of the EBKFs for a small number of ensemble members ranging from two to three in Scenarios S1 and S2. This is because the EBKFs yield inaccurate state estimation results if a small $N_{e}$ is employed, owing to sampling error from a finite ensemble size.

As described in Section 2.1, the posterior error covariance $P_{k|k}$ in the EnKF is systematically underestimated due to the inaccurate calculation of the posterior error covariance $P_{k|k}$ [27]. Accordingly, the estimation performance of the EnKF was degraded by underweighted observations, resulting in more inaccurate positioning results compared to other EBKFs when $N_{e}\geq 8$ for Scenario S1 (Figure 2) and $N_{e}\geq 40$ for Scenario S2 (Figure 3).

As mentioned in Section 2.1, the EnKF-PO treats perturbed observations as random variables to resolve the issue associated with the systematic underestimation of the error covariance $P_{k|k}$ appearing in the EnKF, thus providing better posterior error statistics with a large ensemble size (i.e., when $N_{e}\geq 8$ for Scenario S1 (Figure 2) and $N_{e}\geq 40$ for Scenario S2 (Figure 3)). However, the additional sampling error related to perturbed observations can induce poor estimation accuracy for a small number of ensemble members due to the systematic underestimation (biased estimate) of the error covariances $P_{k|k-1}$ and $P_{k|k}$ [28]. When $N_{e}\leq 4$ for Scenario S1 (Figure 2) and $N_{e}\leq 8$ for Scenario S2 (Figure 3), the EnKF-PO produced higher positioning error compared with other EBKFs.

Unlike the EnKF-PO, the EnSRF uses the reduced Kalman gain to resolve the problem associated with the systematic underestimation of the posterior error covariance $P_{k|k}$ in the EnKF. Hence, it does not result in an extra source of the sampling error relevant to perturbed observations as in the EnKF-PO, thereby yielding lower positioning error than the EnKF-PO for a small ensemble size, as shown in Figure 2 and Figure 3. In addition, Figure 2 and Figure 3 show that the EnSRF can estimate the pedestrian position better than the EnKF.

As the ensemble size $N_{e}$ is increased above the optimal value (i.e., when $N_{e}\geq 8$ for Scenario S1 and $N_{e}\geq 40$ for Scenario S2), the positioning accuracy difference between the EnKF-PO and EnSRF is not significant, as shown in Figure 2 and Figure 3.

The ETKF uses the ensemble transformation to resolve the problem of the systematic underestimation of the error covariances $P_{k|k-1}$ and $P_{k|k}$ appearing in the EnKF and EnKF-PO. However, the ETKF does not take into consideration the model error $w_{k}$ in the state transition (or prediction) model as in Equation (1); that is, it assumes a perfect model without any model error. Therefore, since the error covariances $P_{k|k-1}$ and $P_{k|k}$ estimated by the ensemble spread in the ETKF are blind to the model error covariance $Q_{k}$ as in Equations (5) and (11), they can be systematically underestimated. For this reason, the ETKF produced less accurate positioning results than the EnKF-PO and EnSRF with $N_{e}\geq 8$ for Scenario S1 (Figure 2) and $N_{e}\geq 40$ for Scenario S2 (Figure 3). The results are induced by underweighted observations, owing to the systematic underestimation of the error covariance.

Unlike the ETKF, the QETKF can estimate the error covariances $P_{k|k-1}^{Q}$ and $P_{k|k}^{Q}$ given by Equations (23) and (24) where the model error covariance $Q_{k}$ is included by considering the model error $w_{k}$ in the state transition model as in Equation (22). Therefore, it can avoid the systematic underestimation of the error covariances that appears in the ETKF, producing more inflated ensemble spread than the ETKF (refer to Section 4.1.4). This allows the QETKF to yield higher estimation accuracy than the ETKF. Additionally, Figure 2 and Figure 3 show that the QETKF can offer more accurate localization results than other EBKFs by inflating the ensemble perturbations $X^{a}$ with inflation parameters α and β. However, for a small ensemble size, the QETKF yielded nearly identical results to the EnKF or EnSRF, owing to the sampling error. The positioning accuracy for Bayes filters is addressed in more detail in the next subsection.

#### 4.1.3. Positioning Accuracy and Computational Time

Table 2 and Table 3 compare the overall estimation performance of localization methods PU1, PU2, and PU3 based on Bayes filters (KF and EBKFs using $N_{e}=8$ for Scenario S1 and $N_{e}=40$ for Scenario S2) through the averaged positioning error during experiments executed by fifty users in Scenarios S1 and S2. Empirical experiments (not shown here) showed that localization methods PU1, PU2, and PU3 using both prediction and update phases have lower positioning errors compared with the positioning scheme P (i.e., PDR) using only the prediction phase.

As seen in Table 2 and Table 3, comparing the positioning results of PU1, PU2, and PU3 reveals that the type of RSS signal data used to obtain the observation for the user position in the fingerprinting approach does not have a great impact on the localization performance. From Table 2 and Table 3, we can see that the QETKF can achieve better positioning accuracy compared to other filters. For example, the QETKF provided about 41%, 33%, and 38% lower localization errors than the ETKF for methods PU1, PU2, and PU3 in Scenario S1, respectively (see Table 2). Moreover, it accomplished about 13%, 21%, and 18% lower positioning errors compared with the ETKF for methods PU1, PU2, and PU3 in Scenario S2, respectively (see Table 3).

This is because the model error covariance $Q_{k}$ in the QETKF is added at the end of the error covariances $P_{k|k-1}^{Q}$ and $P_{k|k}^{Q}$ as in Equations (23) and (24) by considering the model error $w_{k}$ in the prediction model given by Equation (22). Therefore, the error covariances $P_{k|k-1}^{Q}$ and $P_{k|k}^{Q}$ estimated by the QETKF are not systematically underestimated, unlike $P_{k|k-1}$ and $P_{k|k}$ estimated by the ETKF. On the contrary, since the ETKF does not take the model error $w_{k}$ into account as in Equation (1), the error covariances $P_{k|k-1}$ and $P_{k|k}$ estimated by it do not include the model error covariance $Q_{k}$ and thereby can be systematically underestimated as in Equations (5) and (11). As a result, the ETKF produced less accurate positioning results than the QETKF by underweighted observations due to the systematic underestimation of the error covariances, as shown in Table 2 and Table 3.

Table 2 and Table 3 also show the average values of the computational time required for each positioning method based on Bayes filters to estimate the user position in Scenarios S1 and S2 during experiments executed by fifty users. From Table 2 and Table 3, we can see that the computational costs from the KF are almost the same as those from the EBKFs, regardless of the localization method in Scenarios S1 and S2. For all the experiments performed in this study, since a small number of observations (i.e., observation vector $z_{k}$ with l=2 that consists of the observations for state variables $x_{k}$ and $y_{k}$ of the pedestrian) were assimilated to estimate the user position, the ETKF and QETKF based on the ensemble transformation provided no significant benefits over other EBKFs for the computational time.

**Figure 4:**
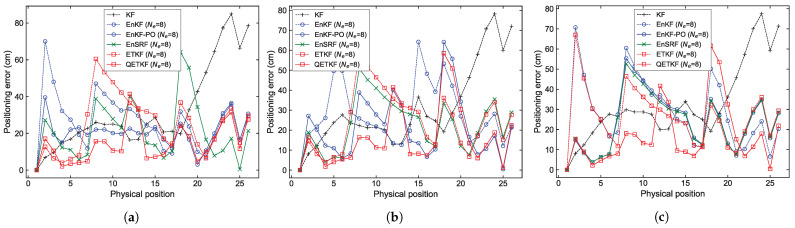
Positioning errors obtained by positioning schemes (**a**) PU1, (**b**) PU2, and (**c**) PU3 based on Bayes filters at physical positions represented by a sequence number in Scenario S1.

**Figure 5:**
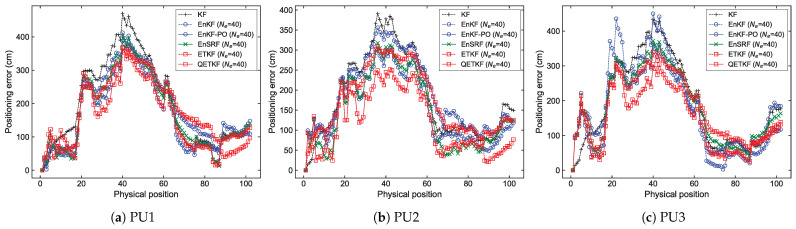
As in Figure 4, but for Scenario S2.

**Figure 6:**
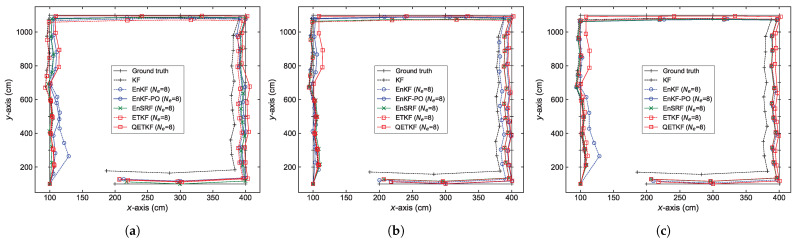
Pedestrian trajectories obtained by positioning schemes (**a**) PU1, (**b**) PU2, and (**c**) PU3 based on Bayes filters at physical positions represented by a sequence number in Scenario S1. The trajectories are denoted using *x*-axis and *y*-axis coordinates.

**Figure 7:**
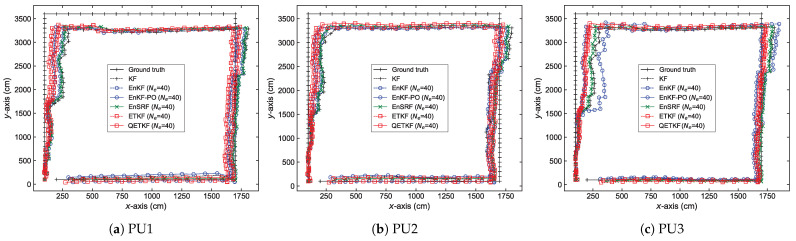
As in Figure 6, but for Scenario S2.

The localization errors can be analyzed in more detail from Figure 4, Figure 5, Figure 6 and Figure 7, which denote average positioning errors (Figure 4 and Figure 5) and pedestrian trajectories (Figure 6 and Figure 7) calculated by localization methods PU1, PU2, and PU3 based on Bayes filters (KF and EBKFs using $N_{e}=8$ for Scenario S1 and $N_{e}=40$ for Scenario S2) at physical positions represented by a sequence number in Scenarios S1 (Figure 4 and Figure 6) and S2 (Figure 5 and Figure 7) during experiments executed by fifty pedestrians.

On average, the use of the EnKF-PO, EnSRF, and QETKF to resolve the problem of the systematic underestimation of the error covariances could provide more accurate positioning results compared to the EnKF and ETKF with the systematic underestimation of the error covariances $P_{k|k-1}$ and $P_{k|k}$, as shown in Figure 4, Figure 5, Figure 6 and Figure 7. The positioning performance of the EBKFs is discussed in more detail through the rank histogram in the next subsection. On average, the QETKF produced better positioning results than other filters by inflating the ensemble perturbations $X^{a}$ with inflation parameters α and β, especially even at physical positions denoted by 20 to 60 in Scenario S2, where RF signals are frequently blocked by walls and obstacles (Figure 5 and Figure 7). The results demonstrate that the QETKF can provide a satisfactory filtering solution, even in a building with many complicated obstacles and poor wireless signals.

#### 4.1.4. Rank Histogram

**Figure 8:**
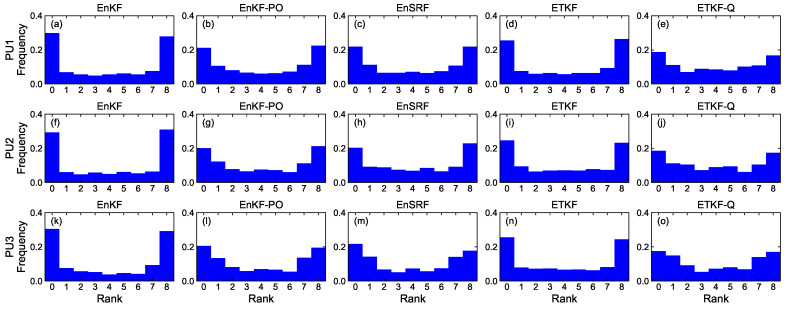
Rank histograms for state variables $x_{k}$ of the pedestrian that are calculated by localization methods PU1, PU2, and PU3 based on EBKFs using $N_{e}=8$ for Scenario S1.

**Figure 9:**
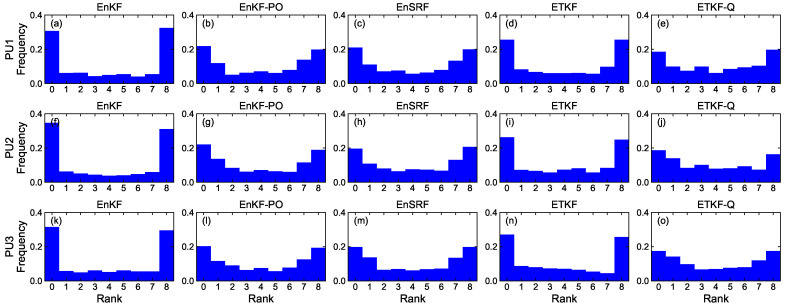
As in Figure 8, but for state variables $y_{k}$.

**Figure 10:**
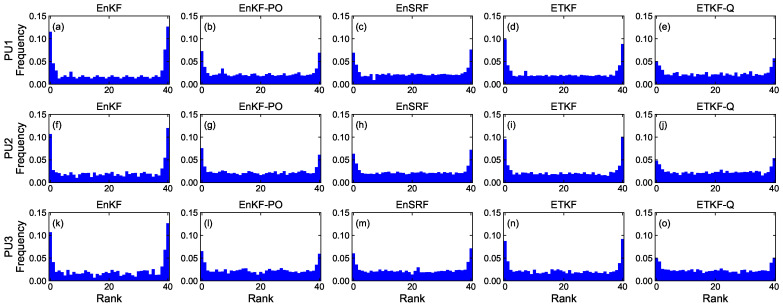
Rank histograms for state variables $x_{k}$ of the pedestrian that are calculated by localization methods PU1, PU2, and PU3 based on EBKFs using $N_{e}=40$ for Scenario S2.

**Figure 11:**
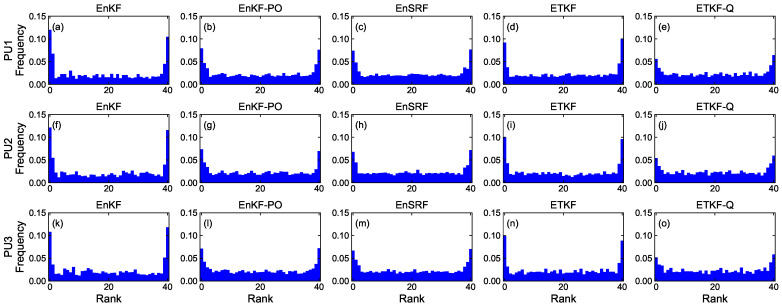
As in Figure 10, but for state variables $y_{k}$.

Figure 8, Figure 9, Figure 10 and Figure 11 denote rank histograms calculated by localization methods PU1, PU2, and PU3 based on EBKFs using $N_{e}=8$ for Scenario S1 and $N_{e}=40$ for Scenario S2 during positioning tests. Rank histograms shown in the figures were generated by repeatedly tallying the rank of the ground truth against values (state variables (*x*-axis and *y*-axis position) of the pedestrian in the navigation frame (i.e., $x_{k}$ and $y_{k}$)) that are estimated from prior ensemble members and then are sorted from lowest to highest [38].

The verification of the rank histogram was executed for state variables $x_{k}$ and $y_{k}$ of the pedestrian using EBKFs with $N_{e}=8$ for Scenario S1 (Figure 8 and Figure 9) and $N_{e}=40$ for Scenario S2 (Figure 10 and Figure 11). On average, the uniform distribution of the rank histogram suggests that the ensemble spread estimated by EBKFs is close to the probability distribution of the ground truth state, thereby providing reliable positioning results. On the contrary, the nonuniform histograms denote that the probabilistic representation by the ensemble members of EBKFs deviates from the probability distribution of the ground truth state, thus yielding inaccurate localization results.

The U-shape rank histograms in Figure 8a,d,f,i,k,n and Figure 9a,d,f,i,k,n represent severe drawbacks to the positioning performance by the EnKF and ETKF with the systematic underestimation of the error covariances $P_{k|k-1}$ and $P_{k|k}$, as shown in Figure 4, Figure 5, Figure 6 and Figure 7. For the EnKF and ETKF, the substantial deviations from the probability distribution of the ground truth come from the inaccurate calculation of the posterior error covariance $P_{k|k}$ [27] and no consideration for model error $w_{k}$, respectively, thereby representing the biased estimate of the ensemble spread.

Figure 8 and Figure 9 also show the advantage of the EnKF-PO, EnSRF, and QETKF, which are designed to resolve issues related to the systematic underestimation of the error covariances $P_{k|k-1}$ and $P_{k|k}$ appearing in the EnKF and ETKF, in providing ensemble spreads closer to the truth than the EnKF and ETKF.

Similar to Figure 8 and Figure 9, the rank histograms in Figure 10 and Figure 11 denote that the EnKF-PO, EnSRF, and QETKF can provide a probabilistic representation by the ensemble members closer to the probability distribution of the ground truth than the EnKF and ETKF, thereby yielding better localization results (Figure 4, Figure 5, Figure 6 and Figure 7).

Nonetheless, as shown in Figure 8, Figure 9, Figure 10 and Figure 11, the nonuniform rank histograms of the EnKF-PO, EnSRF, and QETKF represent that their ensemble spreads still deviate from the probability distribution of the ground truth; that is, their error covariances $P_{k|k-1}$ and $P_{k|k}$ are still underestimated. To resolve this issue, the localization methods will be enhanced to provide ensemble spreads closer to the truth by applying additive and adaptive covariance inflation approaches [39,40] to EBKFs in future work.

### 4.2. Cycling Data Assimilation Experiments

#### 4.2.1. L96 Model

In this section, to examine the validity of the QETKF algorithm as an ensemble data assimilation method, the QEKTF is applied to the L96 model [31], which is commonly used as a simple chaotic dynamical system in ensemble data assimilation [41]. The L96 model uses $N_{x}$ model variables ${\left\{x_{i}\right\}}_{i=1}^{N_{x}}$ that can be regarded as atmospheric variables in $N_{x}$ equally spaced model grid points around a circle of constant latitude. The *i*th variable in the L96 model is propagated in time by the following differential equation with cyclic boundary conditions (i.e., $x_{i+N_{x}}$ and $x_{i-N_{x}}$ are equivalent to $x_{i}$):

(27) $$\frac{dx_{i}}{dt}=\left(x_{i+1}-x_{i-2}\right)x_{i-1}-x_{i}+F.$$

Similar to the L96 model setup used in Ott et al. [41], the number of model variables $N_{x}$ and the external forcing *F* for experiments in this study were set to 40 and 8, respectively. The choice of F=8 leads to chaotic behavior in system dynamics. Assuming that a unit of time Δt=1 is equal to 5 days in the L96 model, the fourth-order Runge–Kutta scheme was used for time integration of Equation (27) with a time step of Δt=0.05 (i.e., 6 h).

#### 4.2.2. Experimental Settings

To examine the model state estimation performance of EBKF algorithms, several data assimilation experiments were executed using the L96 model given by Equation (27) as the state transition function and linear observation operator $h\left(x_{k}\right)=x_{k}$.

Given that model errors $w_{k}$ and observation errors $v_{k}$ are white and uncorrelated, the model error covariance matrix $Q_{k}$ and observation error covariance matrix $R_{k}$ are assumed to be diagonal. For experiments, the diagonal elements of covariance matrices $Q_{k}$ and $R_{k}$ were set to 1 and the tunable parameter β for the QETKF was set to 0.4. True model states were obtained by integrating Equation (27) during 6000 cycles (time steps) using the fourth-order Runge–Kutta scheme with the initial state $x_{0}$, which is determined by adding the model error $w_{k}∼N\left(0,Q_{k}\right)$ to the external forcing *F*. The number of observation variables that are equally spaced on the model grid points was set to 40. The observation $z_{k}$ was obtained by adding the observation error $v_{k}∼N\left(0,R_{k}\right)$ to the true model state using Equation (2). The observation $z_{k}$ was assimilated to the model state every 6 h.

After a spin-up period of 1000 cycles, the model state estimated by the EBKFs and its corresponding root-mean-square error (RMSE) against the true state over 5000 cycles were computed to analyze the assimilation performance of the EBKFs. The cycling experiments were carried out on the web server used for the indoor positioning system (refer to Section 3.2).

#### 4.2.3. Estimation Accuracy and Computational Time

**Figure 12:**
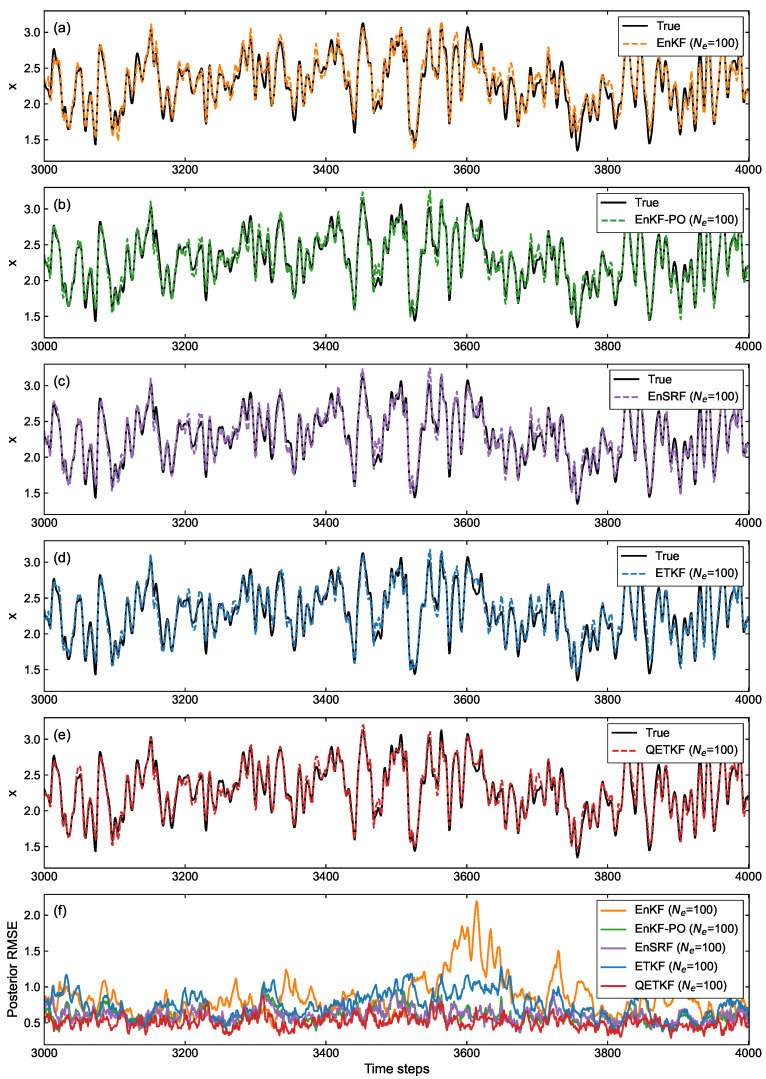
For the L96 model and linear observation operator $h\left(x_{k}\right)=x_{k}$, (**a**–**e**) posterior model state estimates (dashed curves) obtained by EBKF algorithms using $N_{e}=100$ and (**f**) corresponding RMSEs (solid curves) against the true state: (**a**) EnKF, (**b**) EnKF-PO, (**c**) EnSRF, (**d**) ETKF, and (**e**) QETKF. The solid black curves in (**a**–**e**) represent the true state. The posterior state estimation in (**a**–**e**) and its RMSE in (**f**) are averaged over all model variables.

Figure 12 shows the posterior model state estimation (Figure 12a–e) obtained by EBKF algorithms using $N_{e}=100$ and its RMSE (Figure 12f) against the true state over time steps from 3000 to 4000 during cycling experiments for a much clearer performance analysis between the EBKFs. The posterior state estimation and its RMSE in this figure were averaged over all model variables.

On average, due to underweighted observations by the systematic underestimation of the error covariances, the EnKF and ETKF produced less accurate state estimation results than the EnKF-PO, EnSRF, and QETKF to resolve the problem with the systematic underestimation of the error covariances, as shown in Figure 12.

Table 4 shows the posterior RMSE (i.e., actual uncertainty) and estimated error standard deviation referred to as ensemble spread (i.e., estimated uncertainty) given by Equations (18) and (19) that are averaged over all model variables during 5000 time steps after the spin-up period for EBKF algorithms using $N_{e}=100$. From Table 4, we can see that the estimated error standard deviations of the EnKF-PO, EnSRF, and QETKF are closer to the RMSEs than those of the EnKF and ETKF. Therefore, the EnKF-PO, EnSRF, and QETKF can provide better estimation results than the EnKF and ETKF, yielding smaller posterior RMSEs. As seen in Table 4, the QETKF, which inflates the ensemble perturbations $X^{a}$ with inflation parameters α and β, can provide more accurate data assimilation results than other filters.

Table 4 also shows the mean computational time required for EBKF algorithms using $N_{e}=100$ to estimate the model state. As seen in Table 4, the QETKF and ETKF consume less computational time compared to the EnKF, EnKF-PO, and EnSRF. This is because both the QETKF and ETKF that are based on the ensemble transformation performed with the posterior error covariance in ensemble space can provide more rapid estimation results than the EnKF, EnKF-PO, and EnSRF when many observation variables are assimilated as in the atmospheric data assimilation. On the contrary, for indoor user localization experiments in Section 4.1.3, since a small number of observations were assimilated to estimate the user position, the QETKF and ETKF based on the ensemble transformation provided no significant benefits over other EBKFs for the computational time.

## 5. Conclusions

This paper has presented the feasibility of the QETKF as an efficient approach for implementing the ETKF applied to the indoor pedestrian localization system. This study is similar to the previous study [13] in that it estimates the user position by fusing noisy positional information obtained from the PDR and RSS fingerprinting using the Bayes filter in the indoor pedestrian positioning system. However, this study differs from the previous study [13] in that it uses the QETKF as the Bayes filer for the indoor pedestrian positioning instead of the SKPF proposed in the previous study. The QETKF estimates the corrected user position ${\hat{x}}_{k|k}$ by fusing the predicted position ${\hat{x}}_{k|k-1}$ by the prediction step (PDR) and the positional measurement $z_{k}$ estimated by the RSS fingerprinting scheme using the ensemble transformation, while the SKPF computes the updated user position ${\hat{x}}_{k|k}$ by fusing them using both the unscented transformation (UT) of UKF and the weighting method of PF.

Similar to the ETKF widely used to estimate the state of the atmospheric and ocean models, the QETKF estimates the state of the model through the ensemble transformation using the posterior error covariance ${\tilde{P}}^{a}$ in ensemble space. Since the ETKF does not consider the model error $w_{k}$ in the state transition model, the error covariances $P_{k|k-1}$ and $P_{k|k}$ estimated by it can be systematically underestimated. Hence, its positioning performance can be degraded by underweighted observations. To solve the problem, the QETKF considers the model error $w_{k}$ in the state prediction model. Therefore, it can prevent the systematic underestimation of the error covariances $P_{k|k-1}$ and $P_{k|k}$ that appears in the ETKF and can apply the ensemble transformation of the ETKF to the state estimation of the indoor pedestrian model with the model error.

Indoor pedestrian positioning experiments show that the QETKF can produce more accurate positioning results than the ETKF where observations are underweighted due to the systematic underestimation of the error covariances $P_{k|k-1}$ and $P_{k|k}$. In addition, experimental results show that the QETKF can offer better localization results than other EBKFs by inflating the ensemble perturbations $X^{a}$ with inflation parameters α and β, especially even at physical positions where RF signals are frequently blocked by walls and obstacles. The results demonstrate that the QETKF can provide a satisfactory filtering solution, even in a building with many complicated obstacles and poor wireless signals.

Experimental results using rank histograms show that QETKF that is designed to resolve the systematic underestimation of the error covariances can provide ensemble spreads closer to the probability distribution of the ground truth state than the ETKF. This indicates that the QETKF has the potential to become an efficient approach to performing better position estimation with more accurately estimated error covariances for the indoor pedestrian localization system.

Nonetheless, the nonuniform rank histograms of the QETKF suggest that its ensemble spread still deviates from the probability distribution of the ground truth; that is, its error covariances $P_{k|k-1}$ and $P_{k|k}$ are still underestimated. To solve the problem, the ensemble sampling procedure of the QETKF will be improved to produce the ensemble spread that is closer to the truth by using additive and adaptive covariance inflation approaches [39,40] in future work.

Furthermore, cycling experiments executed to verify the data assimilation performance of the QETKF using the L96 model and many observation variables show that the QEKTF can require less computational cost than the EnKF, EnKF-PO, and EnSRF while yielding higher state estimation accuracy than other EBKFs.

For positioning experiments (not reported here), the PF with the optimal number of particles ($N_{e}=10^{3}$) yielded average positioning error values of about 5 cm and 25 cm for Scenarios S1 and S2, respectively (see Figure 12 in [13]), and its average computation time required for positioning methods PU1, PU2, and PU3 in Scenarios S1 and S2 was about 0.1 s. As future work, the positioning performance of the QETKF will be improved to achieve comparable estimation accuracy to the PF while providing higher computational efficiency. Furthermore, positioning experiments using various test sites and MEMS sensors on the smartphone will be the topic of future work that will investigate the positioning accuracy and computational cost of the QETKF as a Bayes filter for indoor pedestrian positioning.

## Figures and Tables

**Table 1:** Localization approaches for indoor positioning experiments.

Notation	Description
P	Position prediction (i.e., PDR) through user direction and acceleration values obtained by accelerometer and gyroscope
PU1	Position prediction by P and position update (correction) through the positional observation $z_{k}$ obtained with user direction and WiFi RSS values
PU2	As in PU1, but with user direction and iBeacon RSS values
PU3	As in PU1, but with user direction, WiFi RSS, and iBeacon RSS values

**Table 2:** Positioning error and computational time of each Bayes filter for Scenario S1.

Bayes Filter	PU1			PU2			PU3	
	Positioning Error (cm)	Computational Time (s)		Positioning Error (cm)	Computational Time (s)		Positioning Error (cm)	Computational Time (s)
KF	32.32	2.41 × 10${}^{-6}$		31.01	2.42 × 10${}^{-6}$		32.76	2.41 × 10${}^{-6}$
EnKF (with $N_{e}=8$)	26.83	2.52 × 10${}^{-6}$		27.69	2.52 × 10${}^{-6}$		28.73	2.51 × 10${}^{-6}$
EnKF-PO (with $N_{e}=8$)	20.24	2.55 × 10${}^{-6}$		21.34	2.54 × 10${}^{-6}$		23.70	2.53 × 10${}^{-6}$
EnSRF (with $N_{e}=8$)	21.29	2.54 × 10${}^{-6}$		22.92	2.53 × 10${}^{-6}$		23.22	2.54 × 10${}^{-6}$
ETKF (with $N_{e}=8$)	24.59	2.52 × 10${}^{-6}$		24.32	2.52 × 10${}^{-6}$		27.38	2.53 × 10${}^{-6}$
QETKF (with $N_{e}=8$)	14.57	2.53 × 10${}^{-6}$		16.31	2.52 × 10${}^{-6}$		17.02	2.52 × 10${}^{-6}$

**Table 3:** Positioning error and computational time of each Bayes filter for Scenario S2.

Bayes Filter	PU1			PU2			PU3	
	Positioning Error (cm)	Computational Time (s)		Positioning Error (cm)	Computational Time (s)		Positioning Error (cm)	Computational Time (s)
KF	199.19	2.41 × 10${}^{-6}$		184.12	2.42 × 10${}^{-6}$		192.78	2.41 × 10${}^{-6}$
EnKF (with $N_{e}=40$)	188.20	2.60 × 10${}^{-6}$		177.48	2.59 × 10${}^{-6}$		182.68	2.59 × 10${}^{-6}$
EnKF-PO (with $N_{e}=40$)	165.59	2.59 × 10${}^{-6}$		164.56	2.58 × 10${}^{-6}$		169.76	2.59 × 10${}^{-6}$
EnSRF (with $N_{e}=40$)	175.65	2.59 × 10${}^{-6}$		147.85	2.62 × 10${}^{-6}$		172.40	2.58 × 10${}^{-6}$
ETKF (with $N_{e}=40$)	180.41	2.59 × 10${}^{-6}$		162.66	2.59 × 10${}^{-6}$		177.94	2.59 × 10${}^{-6}$
QETKF (with $N_{e}=40$)	157.08	2.58 × 10${}^{-6}$		128.43	2.59 × 10${}^{-6}$		145.83	2.59 × 10${}^{-6}$

**Table 4:** Posterior RMSE, ensemble spread (estimated error standard deviation), and computational time of EBKF algorithms using $N_{e}=100$.

EBKF Algorithm	RMSE (Actual Uncertainty)	Ensemble Spread (Estimated Uncertainty)	Computational Time (s)
EnKF ($N_{e}=100$)	0.97	0.45	16.84
EnKF-PO ($N_{e}=100$)	0.65	0.56	17.72
EnSRF ($N_{e}=100$)	0.64	0.57	17.03
ETKF ($N_{e}=100$)	0.83	0.51	14.37
QETKF ($N_{e}=100$)	0.55	0.53	14.95

## Data Availability

All data used in the analysis are freely available online.

## Appendix A. Posterior Error Covariance for the QETKF

In the EnKF algorithm, the optimal Kalman gain $K_{k}$ in Equation (9) can be rewritten as in [42]:

(A1) $$K_{k}=P_{\mathrm{xz}}S_{k}^{-1}$$

where $P_{\mathrm{xz}}$ is the cross covariance between ${\hat{x}}_{k|k-1}$ and ${\hat{z}}_{k}$ and is determined by

(A2) $$P_{\mathrm{xz}}={\left(n-1\right)}^{-1}X^{b}{\left(Y^{b}\right)}^{T}.$$

From Equations (A1) and (A2), the posterior ensemble error covariance $P_{k|k}$ in Equation (11) can be rewritten as

(A3) $$\begin{array}{cc} P_{k|k} & =P_{k|k-1}-K_{k}S_{k}K_{k}^{T}\\ & =P_{k|k-1}-K_{k}P_{\mathrm{xz}}^{T}. \end{array}$$

By replacing the prior error covariance $P_{k|k-1}$ in Equation (A3) with Equation (23), Equation (24) can be derived as

(A4) $$\begin{array}{cc} P_{k|k}^{Q} & =P_{k|k-1}^{Q}-K_{k}P_{\mathrm{xz}}^{T}\\ & ={\left(n-1\right)}^{-1}X^{b}{\left(X^{b}\right)}^{T}+Q_{k}-X^{b}{\left[\left(n-1\right)I+{\left(Y^{b}\right)}^{T}R_{k}^{-1}Y^{b}\right]}^{-1}{\left(Y^{b}\right)}^{T}R_{k}^{-1}{\left(n-1\right)}^{-1}Y^{b}{\left(X^{b}\right)}^{T}\\ & ={\left(n-1\right)}^{-1}X^{b}\left\{I-{\left[\left(n-1\right)I+{\left(Y^{b}\right)}^{T}R_{k}^{-1}Y^{b}\right]}^{-1}{\left(Y^{b}\right)}^{T}R_{k}^{-1}Y^{b}\right\}{\left(X^{b}\right)}^{T}+Q_{k}\\ & ={\left(n-1\right)}^{-1}X^{b}\left\{I-{\left(n-1\right)}^{-1}{\left[I+{\left(n-1\right)}^{-1}{\left(Y^{b}\right)}^{T}R_{k}^{-1}Y^{b}\right]}^{-1}{\left(Y^{b}\right)}^{T}R_{k}^{-1}Y^{b}\right\}{\left(X^{b}\right)}^{T}+Q_{k}\\ & ={\left(n-1\right)}^{-1}X^{b}\left\{I-{\left[I+{\left(n-1\right)}^{-1}{\left(Y^{b}\right)}^{T}R_{k}^{-1}Y^{b}\right]}^{-1}{\left(n-1\right)}^{-1}{\left(Y^{b}\right)}^{T}R_{k}^{-1}Y^{b}\right\}{\left(X^{b}\right)}^{T}+Q_{k}. \end{array}$$

Using the identity $I-{\left(I+A\right)}^{-1}A={\left(I+A\right)}^{-1}$ with $A={\left(n-1\right)}^{-1}{\left(Y^{b}\right)}^{T}R_{k}^{-1}Y^{b}$, Equation (A4) can be expressed as

(A5) $$\begin{array}{cc} P_{k|k}^{Q} & ={\left(n-1\right)}^{-1}X^{b}\left[I-{\left(I+A\right)}^{-1}A\right]{\left(X^{b}\right)}^{T}+Q_{k}\\ & ={\left(n-1\right)}^{-1}X^{b}{\left(I+A\right)}^{-1}{\left(X^{b}\right)}^{T}+Q_{k}\\ & ={\left(n-1\right)}^{-1}X^{b}{\left[I+{\left(n-1\right)}^{-1}{\left(Y^{b}\right)}^{T}R_{k}^{-1}Y^{b}\right]}^{-1}{\left(X^{b}\right)}^{T}+Q_{k}\\ & =X^{b}{\left[\left(n-1\right)I+{\left(Y^{b}\right)}^{T}R_{k}^{-1}Y^{b}\right]}^{-1}{\left(X^{b}\right)}^{T}+Q_{k}\\ & =X^{b}{\tilde{P}}^{a}{\left(X^{b}\right)}^{T}+Q_{k}. \end{array}$$

The use of Equation (A5) enables the QETKF to avoid the systematic underestimation of the posterior error covariance appearing in the ETKF, as in Equation (11).
